# Yale Food Addiction Scale 2.0 (YFAS 2.0) and modified YFAS 2.0 (mYFAS 2.0): Rasch analysis and differential item functioning

**DOI:** 10.1186/s40337-022-00708-5

**Published:** 2022-11-28

**Authors:** Mohsen Saffari, Chia-Wei Fan, Yen-Ling Chang, Po-Ching Huang, Serene En Hui Tung, Wai Chuen Poon, Chien-Ching Lin, Wen-Chi Yang, Chung-Ying Lin, Marc N. Potenza

**Affiliations:** 1 Health Research Center, Life Style Institute, Baqiyatallah University of Medical Sciences, Tehran, Iran; 2 Health Education Department, Faculty of Health, Baqiyatallah University of Medical Sciences, Tehran, Iran; 3 Department of Occupational Therapy, AdventHealth University, Orlando, FL USA; 4 Department of Family Medicine, Cardinal Tien Hospital, New Taipei, Taiwan; 5 Institute of Allied Health Sciences, College of Medicine, National Cheng Kung University, No. 1, University Rd., East Dist., Tainan, 701401 Taiwan; 6 Division of Nutrition and Dietetics, School of Health Sciences, International Medical University, Kuala Lumpur, 57000 Malaysia; 7 Sunway Business School, Sunway University, No. 5, Jalan Universiti, 47500 Bandar Sunway, Selangor Darul Ehsan Malaysia; 8 Department of Laboratory Medicine, National Taiwan University Hospital, Taipei, Taiwan; 9 Division of Hematology and Internal Medicine, National Taiwan University Hospital, Taipei, Taiwan; 10 Graduate Institute of Clinical Medicine, National Taiwan University, Taipei, Taiwan; 11 Infinite Power, Lt. Co., No. 38, Yonghe 1st St., Renwu Dist., Kaohsiung, 814 Taiwan; 12 Faculty of School of Medicine, College of Medicine, I-Shou University, Kaohsiung, 824 Taiwan; 13 Department of Occupational Therapy, College of Medicine, National Cheng Kung University, No. 1, University Rd., East Dist., Tainan, 701401 Taiwan; 14 Department of Public Health, National Cheng Kung University Hospital, College of Medicine, National Cheng Kung University, No. 1, University Rd., East Dist., Tainan, 701401 Taiwan; 15 Biostatistics Consulting Center, National Cheng Kung University Hospital, College of Medicine, National Cheng Kung University, No. 1, University Rd., East Dist., Tainan, 701401 Taiwan; 16 Department of Psychiatry, Yale School of Medicine, New Haven, CT USA; 17 Connecticut Mental Health Center, New Haven, CT USA; 18 Connecticut Council on Problem Gambling, Wethersfield, CT USA; 19 Child Study Center, Yale School of Medicine, New Haven, CT USA; 20 Department of Neuroscience, Yale University, New Haven, CT USA; 21 Wu Tsai Institute, Yale University, New Haven, CT USA; 22 Institute of Allied Health Sciences, Departments of Occupational Therapy and Public Health, and Biostatistics Consulting Center, National Cheng Kung University Hospital, College of Medicine, National Cheng Kung University, No. 1, University Rd., East Dist., Tainan, 701401 Taiwan

**Keywords:** Food addiction, Addictive behaviors, Differential item functioning, Rasch, Validity, Young adults

## Abstract

**Background:**

Food addiction (FA) is a prevalent concern that may manifest as poorly controlled food consumption and promote overweight/obesity. Thus, having a well-established instrument for assessment may facilitate better prevention and treatment. The current study investigated the psychometric properties of two common measures of FA (i.e., the Yale Food Addiction Scale [YFAS] 2.0 and its modified version, mYFAS 2.0) using a robust statistical analysis (Rasch model).

**Methods:**

In this cross-sectional study, the scales were sent to 974 students studying in higher education (60% females) in Taiwan through online media including email and social networks. Rasch modeling was used to assess dimensionality, difficulty level, and item misfit and hierarchy. Differential item functioning (DIF) was performed to examine consistency of the items across gender and weight status.

**Results:**

Rasch analysis indicated 3 items of the 35 items belonging to the YFAS 2.0 (8.6%) and none belonging to the mYFAS 2.0 were misfit. Unidimensionality and construct validity of both scales were supported by appropriate goodness-of-fit for diagnostic criteria. The person separation was 3.14 (reliability = 0.91) for the YFAS 2.0 and 2.17 (reliability = 0.82) for mYFAS 2.0, indicating the scales could distinguish participants into more than 3 strata. Only one substantial DIF was found for diagnostic criteria of “Failure to fulfill major role obligation” in the YFAS 2.0 across gender.

**Conclusion:**

According to Rasch modeling, both the YFAS 2.0 and mYFAS 2.0 have acceptable construct validity in Chinese-speaking youth. Scoring methods using either diagnostic criteria or symptom counts for both the YFAS 2.0 and mYFAS 2.0 are supported by the present Rasch findings.

## Plain English summary

Food addiction is related to eating disorders and may overlap with a variety of disorders, including binge-eating disorder, night-eating syndrome, bulimia nervosa or other conditions. Therefore, it is important for healthcare providers to assess food addiction and one commonly used method is using the Yale Food Addiction Scale (YFAS) developed by Gearhardt and her colleagues. The YFAS has been updated and revised into two versions: the YFAS 2.0 and modified YFAS 2.0 (i.e., mYFAS 2.0). Psychometric testing studies have reported the feasibility and adequate properties for both the YFAS 2.0 and mYFAS 2.0. However, prior studies’ findings were based on classical test theory (CTT) findings. The present study thus used a modern test theory (i.e., Rasch models) to examine if both the YFAS 2.0 and mYFAS 2.0 have similarly satisfactory psychometric properties shown in the CTT findings. The present findings using Rasch models support the use of both the YFAS 2.0 and mYFAS 2.0 to assess food addiction among youth. Therefore, healthcare providers may use either the YFAS 2.0 or mYFAS 2.0 to assess levels of food addiction.

## Introduction

Food addiction (FA) has received considerable attention in both laboratory and clinical research [1]. This concept refers to the idea that some foods, especially those with dense calories, heavy processing or high palatability, may promote addictive consumption. FA may be considered as a kind of behavioral addiction (related to eating) or an eating problem which may not constitute a psychiatric disorder. FA may overlap with binge-eating disorder, night-eating syndrome, bulimia nervosa or other conditions [2, 3]. A hypothesis that an addictive process related to neural features may contribute to excessive eating is an underlying conceptual feature of FA [4]. However, FA is not classified as a formal diagnosis in the fifth edition of the Diagnostic and Statistical Manual of Mental Disorders (DSM-5), although it has been discussed as a possible psychiatric disorder [5]. People with FA often express symptoms such as considerable distress in relation to specific foods, eating more food than planned, eating more than needed to relieve hunger, feelings of lost control over food intake, unsuccessful attempts to reduce eating particular foods, and diminished interests in participating in some experiences due to fear of overeating [6, 7]. FA has been associated with multiple mental disorders including anxiety, depressive, attention-deficit/hyperactivity, post-traumatic stress and binge-eating disorders [7].

To date, there are limited data on the prevalence of FA globally. However, some general-population studies suggest that between 4 and 10% of people may experience FA, and it is more prevalent in females than males [2, 3]. Its prevalence among people with overweight or those seeking weight-loss may range between 16 and 30%, higher than in general populations [8]. A study conducted in college students in China revealed that nearly 7% of participants may have experienced mild to severe FA [9]. However, since nearly two-thirds of the world population has overweight/obesity, the role of FA as a contributor should be investigated and addressed [10]. The prevalence of obesity in China has risen from 3 to 8% during the last decade, and currently more than 90 million people live with obesity in this country [11].

Having a psychometrically sound instrument to measure FA is important for detection and timely intervention [12]. Historically, few instruments have been available to assess addictive eating behaviors, and most were not comprehensive for evaluating different aspects of addictive eating tendencies [13]. Consequently, the Yale Food Addiction Scale (YFAS) was developed to address this concern and revealed acceptable psychometric properties across different translations into languages including French, Italian, Persian, Chinese and Turkish [13–17]. An updated version of the YFAS (i.e., YFAS 2.0) was published in 2016. The YFAS 2.0 included four essential criteria to diagnose FA based on DSM-5-related criteria for substance-use disorders [18]. These included craving, consumption despite negative social/ interpersonal consequences, failure to perform role obligations, and consumption in physically dangerous settings [5, 18].

The YFAS 2.0 is a commonly employed measure of FA and has 35 items that assess 11 indicators of addictive behaviors, distress, and related clinical impairment [3]. An abbreviated version of this measure, the modified YFAS 2.0 (mYFAS 2.0), includes 13 items and is available for use as a short screening measure to assess FA [19]. Although, perhaps due to fewer questions, the mYFAS 2.0 is a less sensitive instrument than the YFAS 2.0 to measure addictive eating, it has demonstrated appropriate validity and reliability in several studies [9, 19, 20]. Having a valid and reliable instrument with fewer questions may help reduce burden on respondents during the screening process and can be time-saving for both participants and researchers [14]. Nevertheless, both scales currently serve as standard measures of FA with relatively similar results.

Although these instruments have been validated in many languages with acceptable psychometric properties, the validation process was mostly done based on traditional approaches using classical test theory (CTT) methods. In this approach, the quality of an item is assessed by the degree of the association between participants’ response pattern for that item and their scores for all items [21]. However, there are some shortcomings to this approach including test and sample size dependence, considering equal weights for all items while there may be differences in the difficulty levels between items, and using a constant standard error of measurement and ordinal values to compute total scores [22, 23]. These factors may influence accurate measurements. In contrast, the Rasch model applies a modern item response theory and has been recognized as a gold standard in validation processes. Thus, it may resolve many CTT-related shortcomings [21].

The Rasch model allows researchers to critically evaluate scales using parametric tests by transforming categorical data into quantitative data [24]. In the Rasch model, a scale is examined against a mathematical measurement model that clarifies what should be in the item responses using interval-based measures. The interval data versus ordinal values provide more robust and accurate findings on the structural validity and objectivity of the scale [21]. The model contains more quantitative information and a continuous scale of measurement compared to a CTT approach and assumes that each individual has a fixed latent tendency along with each item with a particular fixed difficulty [25]. The Rasch model also will help assess the unidimensionality of both the YFAS 2.0 and mYFAS 2.0, and both instruments have been found to be unidimensional [20, 26–38]. Moreover, both the YFAS 2.0 and mYFAS 2.0 were expected to create a single factor structure of FA to differentiate between those with or without FA [18, 19]. Therefore, we may confirm this feature by Rasch model indicating the appropriateness of the scale for such measurements. Therefore, examination of the factor structure of this scale using traditional methods like exploratory factor analysis may not be particularly helpful.

The differential item functioning (DIF) or item bias in subsamples also may be assessed using Rasch analysis. The presence of DIF suggests that the likelihood of a correct response among people who are assumed to be test-taking with equal abilities, in subgroups based on gender, race/ethnicity, income and other variables, may be different. Thus, DIF provides negative evidence for the validity of a scale across groups [24, 39].

A psychometrically sound scale should ideally be examined using various statistical techniques to provide greater empirical evidence supporting its validity [12]. To the best of our knowledge, Rasch analysis has not been previously used to assess the YFAS 2.0 and mYFAS 2.0. Thus, the current study aimed to assess the psychometric properties of the scales using this modern approach. Further, differential responses among groups based on gender and body mass index (BMI) were investigated with the hypotheses that both scales would demonstrate validity across groups.

## Methods

### Recruitment procedure for the online survey

The corresponding author (C-YL) sought assistance from his university students and faculty members to spread information about this online survey. The university students and faculty members were instructed to send the online survey information via multiple forums (e.g., *LINE*, *Facebook*, email, or online posts), and the faculty members were informed that they themselves were not the target population to participate in the survey. The online survey was designed in *Google Forms* and all survey items were set to be compulsory to avoid missing answers. Participants were informed that if they completed the survey and provided contact information, each participant could receive 100 New Taiwan Dollars (around 3.3 USD) as an incentive. Before initiating data collection via the online survey, the study was approved by the Human Research Ethics Committee in the National Cheng Kung University (Approval No.: NCKU HREC-E-109-551-2) and the Institutional Review Board in the Chi Mei Medical Center (IRB Serial No.: 11007-006).

### Participants

Target participants in the current study were university students who were enrolled in an undergraduate (including bachelor’s degrees) or a postgraduate program (including master’s and doctoral degrees) in Taiwan when they completed the online survey. The inclusion criteria were (i) having the ability to read and understand online questionnaires written in traditional Chinese characters; (ii) being aged 20 years or above with the ability to provide consent for participation; (iii) not having any psychiatric disorder based on their self-report. Every respondent was requested to provide an e-form informed consent to indicate his or her willingness to participate in the current study. Information regarding the current study and participation rights was described in the first page of the online survey before the participants could click an icon (*agree* or *disagree*) to indicate their willingness to participate.

### Instruments

Both the Yale Food Addiction Scale 2.0 (YFAS 2.0) and modified YFAS 2.0 (mYFAS 2.0) were used. Moreover, the YFAS 2.0 and mYFAS 2.0 were Chinese versions in the present study. Each item in the self-reported YFAS 2.0 and mYFAS 2.0 asks participants about their eating behaviors in the past year, using an 8-point Likert scale (0 = Never; 1 = Less than monthly; 2 = Once a month; 3 = 2–3 times a month; 4 = Once a week; 5 = 2–3 times a week; 6 = 4–6 times a week; 7 = Every Day). The YFAS 2.0 includes 35 symptom items which can be categorized into 11 symptom criteria (33 items) and 1 clinical impairment criterion (2 items). Except for the two clinical significance items, scores can be summed to calculate the 11 symptom criteria. In the mYFAS 2.0, only one item was selected from each of the 11 symptom criteria; along with the 2 clinical impairment items, the mYFAS 2.0 has 13 items. To determine diagnostic thresholds, a threshold for each item was established to each calculate participant’s severity level. Participants can be categorized into no (1 or fewer symptoms or does not meet criteria for clinical impairment), mild (2 or 3 symptoms and clinical impairment), moderate (4 or 5 symptoms and clinical impairment) or severe (6 or more symptoms and clinical impairment FA.

Demographic data including age, gender, level of education, marital status, monthly income, tobacco smoking, alcohol use and information to compute BMI (i.e., height and weight) were collected from participants. BMI was calculated using SPSS software.

### Data analysis

In the current study, we used Rasch analysis to examine the psychometric properties of both the YFAS 2.0 (35 items) and mYFAS 2.0 (13 items). Specifically, we used Facets software to evaluate item fit to investigate test unidimensionality. DIF across subgroups (i.e., gender and BMI status) was examined.

Previous studies have validated that the one-factor model outperformed other models for both the YFAS 2.0 [18] and mYFAS 2.0 [19]. Therefore, all items were examined together assuming one underlying trait in Rasch analyses. Additionally, as the YFAS 2.0 and mYFAS 2.0 can be scored in two manners (i.e., symptom counts and diagnostic criteria), we analyzed their psychometric properties using both options. First, each symptom item can be summed to calculate the symptom counts (ranging from 0–35 and 0–13 in the YFAS 2.0 and mYFAS 2.0, respectively). Second, diagnostic criteria were evaluated according to pre-determined thresholds of the sum of the corresponding symptoms to determine FA severity (i.e., no, mild, moderate, and severe FA). Symptom counts were evaluated with a rating scale Rasch model (rating 0–7) while the diagnostic criteria were evaluated with the dichotomous Rasch model (rating 0 or 1). The test unidimensionality of the YFAS 2.0 and mYFAS 2.0 were examined with goodness-of-fit statistics: items with an infit mean square (MnSq) above 1.5 associated with a standard deviation above 2 were considered misfit [40]. If mean square statistics are acceptable, the standardized score (Zstd) can be ignored. However, if an item has an infit MnSq > 1.5, it indicates a deviation from unidimensionality and should be revised or removed from the scale [41]. We expected that less than 5% of the items in both the YFAS 2.0 and mYFAS 2.0 would fail to meet the criterion [42]. Person separations were also evaluated to determine whether items in the YFAS 2.0 and mYFAS 2.0 distinguished enrolled participants with different levels of FA. A minimum person separation of 2 was expected [43]. Furthermore, Rasch analysis was also used to investigate the hierarchy of items in the YFAS 2.0 and mYFAS 2.0.

We examined the DIF (the extent to which the item hierarchies were inconsistent across groups) defined by gender (female vs. male) and BMI status (BMI ≥ 24 vs. BMI < 24 based on Taiwan norms) [44] to detect potential interactions between subgroups that might generate underlying bias in both the YFAS 2.0 and mYFAS 2.0. We computed the Rasch-Welch *t*-statistics to identify items that exhibited statistically significant DIF (*p* < .05). DIF contrasts less than 0.5 logit were considered negligible; contrasts between 0.5 and 1 logit were considered moderate; and contrasts over 1 were defined as substantial [45]. In order to support unidimensionality, we expected to have no more than 5% of the diagnostic criteria demonstrate substantial DIF [46]. Specifically, when detecting DIF within an assessment, a common practice is to use the item measure calibration derived from the Rasch analysis to yield a reference composite of the potential underlying secondary dimension [47]. When an item measures at least one secondary dimension (in addition to the main latent construct that the assessment was intended to measure) and two groups of participants differ in their underlying ability distribution of the secondary dimensions, then DIF occurs [48]. Therefore, ensuring that there was no DIF or limited DIF items within the YFAS 2.0 and mYFAS 2.0 is important to confirm the measurement unidimensionality. In this case, we expected that there would be less than 2 items with DIF on the YFAS 2.0 and zero to one item on the mYFAS 2.0 across the subgroups. Facets Version 3.84.0 was used to perform the Rasch analysis. Other descriptive statistics were conducted using IBM SPSS 28.0.

## Results

### Demographics

There were 974 participants enrolled. Among them, 578 (59.3%) were female. The mean age was 23.7 years (S.D. = 4.3), and the average BMI was 22.4 (S.D. = 3.6). Most participants were college students (69.7%; the remaining 30.3% were in other types of higher education programs such as master’s or doctoral programs), single (92.7%), non-smoking (69.6%) and non-drinking (65.3%). Detailed demographics can be found in Table 1.

**Table 1:** Participant demographics (N = 974)

Variables	Mean (S.D.) or n (%)
Age	23.7 (4.3)
Height (cm)	167.2 (50.3)
Weight (kg)	63.5 (34.4)
BMI	22.4 (3.6)
*Gender*	
Male	396 (40.7)
Female	578 (59.3)
*Educational level*	
College	679 (69.7)
Master’s	262 (26.9)
Doctoral	26 (2.7)
Others	7 (0.7)
*Marital status*	
Single	903 (92.7)
Married	49 (5.0)
Divorced	5 (0.5)
Separated	1 (0.1)
Others	16 (1.6)
*Monthly income (NTD)*	
Below $4999	140 (14.4)
$5000–9999	99 (10.2)
$10,000–14,999	130 (13.3)
$15,000–19,999	46 (4.7)
$20,000–24,999	98 (10.1)
$25,000–29,999	75 (7.7)
$30,000–34,999	49 (5.0)
$35,000–39,999	22 (2.3)
$40,000–44,999	23 (2.4)
$45,000–49,999	9 (0.9)
Above $50,000	29 (3.0)
Missing	254 (26.1)
*Smoke*	
No	678 (69.6)
Yes	42 (4.3)
Missing	254 (26.1)
*Use alcohol*	
No	636 (65.3)
Yes	84 (8.6)
Missing	254 (26.1)

S.D. standard deviation; *NTD* New Taiwan Dollar; 1 NTD = 0.034 United States Dollar

#### YFAS 2.0

When examining the 35 YFAS 2.0 FA items, Rasch results showed that 3 of the 35 items (3/35 = 8.6%) misfit the Rasch expected values of MnSq and Zstd (Table 2). Two items (#1, #2) from the “*Substance taken in larger amount and for longer period then intended*” category and one item (#7) from the “*Much time/activity to obtain, use, recover*” category misfit, which exceeded the 5% threshold. The three most adopted items among the 35 symptoms were: item #2 “I continued to eat certain foods even though I was no longer hungry,” item #1 “When I started to eat certain foods, I ate much more than planned,” and item #15 “When I cut down or stopped eating certain foods, I had strong cravings for them.” The three least adopted items were: item #21 “I avoided social situations because people wouldn’t approve of how much I ate,” item # 34 “I was so distracted by thinking about food that I could have been hurt (e.g., when driving a car, crossing the street, operating machinery),” and item #33 “I was so distracted by eating that I could have been hurt (e.g., when driving a car, crossing the street, operating machinery).” The measure logits ranged from − 0.95 to 0.42. Table 2 offers additional details.

**Table 2:** Rasch analyses of the 35 YFAS2 symptoms

Item		Measure		SE		Infit				Outfit			
						MnSq﻿		Zstd		MnSq﻿		Zstd	
1. When I started to eat certain foods, I ate much more than planned+		− 0.86		0.03		2.56		9.0		3.25		9.0	
2. I continued to eat certain foods even though I was no longer hungry+		− 0.95		0.03		2.58		9.0		3.33		9.0	
3. I ate to the point where I felt physically ill		− 0.08		0.03		1.14		2.5		1.60		7.2	
4. I worried a lot about cutting down on certain types of food, but I ate them anyways		− 0.15		0.03		1.27		4.7		1.42		5.4	
5. I spent a lot of time feeling sluggish or tired from overeating.		0.03		0.03		0.86		− 2.8		0.81		− 2.7	
6. I spent a lot of time eating certain foods throughout the day		− 0.05		0.03		0.88		− 2.3		0.94		− 0.7	
7. When certain foods were not available, I went out of my way to get them. For example, I went to the store to get certain foods even though I had other things to eat at home+		− 0.43		0.03		1.54		9.0		2.24		9.0	
8. I ate certain foods so often or in such large amounts that I stopped doing other important things. These things may have been working or spending time with family or friends		0.11		0.03		0.81		− 3.8		0.70		− 4.4	
9. I had problems with my family or friends because of how much I overate.		0.21		0.04		0.81		− 3.7		0.65		− 5.1	
10. I avoided work, school or social activities because I was afraid I would overeat there		0.25		0.04		0.80		− 3.8		0.64		− 5.1	
11. When I cut down on or stopped eating certain foods, I felt irritable, nervous or sad		− 0.04		0.03		0.75		− 5.1		0.67		− 5.1	
12. If I had physical symptoms because I hadn’t eaten certain foods, I would eat those foods to feel better		− 0.13		0.03		0.84		− 3.1		0.90		− 1.4	
13. If I had emotional problems because I hadn’t eaten certain foods, I would eat those foods to feel better		− 0.23		0.03		1.00		0		1.06		0.9	
14. When I cut down on or stopped eating certain foods, I had physical symptoms. For example, I had headaches or fatigue		0.18		0.03		0.88		− 2.2		0.95		− 0.6	
15. When I cut down or stopped eating certain foods, I had strong cravings for them		− 0.52		0.03		1.47		8.2		2.06		9.0	
16. My eating behavior caused me a lot of distress		0.08		0.03		0.86		− 2.7		0.84		− 2.2	
17. I had significant problems in my life because of food and eating. These may have been problems with my daily routine, work, school, friends, family, or health		0.11		0.03		0.76		− 4.9		0.75		− 3.5	
18. I felt so bad about overeating that I didn’t do other important things. These things may have been working or spending time with family or friends		0.30		0.04		0.69		− 6.0		0.54		− 6.9	
19. My overeating got in the way of me taking care of my family or doing household chores		0.37		0.04		0.78		− 4.1		0.58		− 6.0	
20. I avoided work, school or social functions because I could not eat certain foods there		0.32		0.04		0.80		− 3.7		0.66		− 4.7	
21. I avoided social situations because people wouldn’t approve of how much I ate		0.42		0.04		0.85		− 2.6		0.65		− 4.6	
22. I kept eating in the same way even though my eating caused emotional problems		0.14		0.03		1.00		0.0		1.01		0.1	
23. I kept eating the same way even though my eating caused physical problems		0.13		0.03		0.89		− 2.0		0.85		− 2.0	
24. Eating the same amount of food did not give me as much enjoyment as it used to		0.16		0.03		0.87		− 2.5		0.75		− 3.5	
25. I really wanted to cut down on or stop eating certain kinds of foods, but I just couldn’t		− 0.16		0.03		1.07		1.2		1.08		1.1	
26.I needed to eat more and more to get the feelings I wanted from eating. This included reducing negative emotions like sadness or increasing pleasure		− 0.13		0.03		0.85		− 2.9		0.95		− 0.7	
27. I didn’t do well at work or school because I was eating too much		0.30		0.04		0.80		− 3.8		0.59		− 6.0	
28. I kept eating certain foods even though I knew it was physically dangerous. For example, I kept eating sweets even though I had diabetes. Or I kept eating fatty foods despite having heart disease		− 0.11		0.03		1.14		2.5		1.19		2.5	
29. I had such strong urges to eat certain foods that I couldn’t think of anything else		0.15		0.03		0.68		− 6.6		0.62		− 5.7	
30. I had such intense cravings for certain foods that I felt like I had to eat them right away		− 0.09		0.03		0.89		− 2.0		0.93		− 0.9	
31. I tried to cut down on or not eat certain kinds of food, but I wasn’t successful		− 0.15		0.03		1.08		1.5		1.11		1.5	
32. I tried and failed to cut down on or stop eating certain foods		− 0.17		0.03		1.11		2.0		1.03		0.4	
33. I was so distracted by eating that I could have been hurt (e.g., when driving a car, crossing the street, operating machinery).		0.38		0.04		0.86		− 2.6		0.60		− 5.5	
34. I was so distracted by thinking about food that I could have been hurt (e.g., when driving a car, crossing the street, operating machinery)		0.40		0.04		0.86		− 2.4		0.61		− 5.4	
35.My friends or family were worried about how much I overate		0.20		0.04		1.15		2.5		1.09		1.0	

*MnSq* mean square; *Zstd* standardized score

+Infit statistics > 1.5 associated with Zstd > 2: item misfit

When examining the goodness-of-fit of the diagnostic criteria from the YFAS 2.0, all 11 met the Rasch expectation with no misfit items (Table 3). These findings confirmed the unidimensionality and supported the construct validity of the YFAS 2.0 when applying the diagnostic criteria. Given the results, it is suggested to use the diagnostic criteria instead of the raw symptom counts. The most adopted criterion was “*Continued use despite social or interpersonal problems*” while the least adopted criterion was “*Much time/activity to obtain, use, recover.*” The measure logits ranged from − 2.46 to 2.29. Please refer to Table 3 for additional details.

**Table 3:** Rasch analyses of the 11 YFAS2 diagnostic criteria

Diagnostic criteria	Measure	SE	Infit		Outfit	
			MnSq	Zstd	MnSq	Zstd
Substance taken in larger amount and for longer period than intended (#1+#2+#3)	.74	.14	1.37	3.9	3.29	8.3
Persistent desire or repeated unsuccessful attempts to quit (#4+#25+#31+#32)	1.36	.15	1.05	.5	2.44	4.3
Much time/activity to obtain, use, recover (#5+#6+#7)	2.29	.17	1.32	2.8	3.10	3.6
Important social, occupational, or recreational activities given up or reduced (#8+#10+#18+#20)	− 2.12	.12	.72	− 5.5	1.12	.6
Use continues despite knowledge of adverse consequences (e.g., emotional problems, physical problems) (#22+#23)	.93	.14	.90	− 1.1	1.44	2.0
Tolerance (marked increase in amount; marked decrease in effect) (#24+#26)	1.69	.16	.85	− 1.6	1.25	.9
Characteristic withdrawal symptoms; substance taken to relieve withdrawal (#11+#12+#13+#14+#15)	− .13	.12	.98	− .3	1.49	3.4
Continued use despite social or interpersonal problems (#9+#21+#35)	− 2.46	.12	.83	− 3.0	.96	.0
Failure to fulfill major role obligation (e.g., work, school, home) (#19+#27)	− 1.88	.12	.84	− 3.1	1.35	1.7
Use in physically hazardous situations (#28+#33+#34)	− 1.72	.11	.82	− 3.5	.87	− .7
Craving, or a strong desire or urge to use (#29+#30)	1.31	.15	.70	− 3.5	.54	− 2.3

*MnSq* mean square; *Zstd* standardized score; *SE* standard error

The person separation for the YFAS 2.0 was 3.14, which is associated with a person reliability of 0.91. These values indicated that the YFAS 2.0 items could distinguish enrolled participants with different levels of FA and exceeded the minimum person separation value expectation of 2, which resulted in 4.52 strata of respondents.

Substantial gender-related DIF was found for one of the 11 diagnostic criteria: “Failure to fulfill major role obligation (e.g., work, school, home).” The probability of item endorsement for this particular criterion was higher for male participants than female participants. The contrasts for criteria by gender (i.e., male vs. female) ranged from − 0.70 to 1.12. No BMI-related DIF was detected in the YFAS 2.0. The contrasts for criteria by BMI status (i.e., BMI ≥ 24 vs. BMI < 24) ranged from − 0.45 to 0.39. Please refer to Table 4 for more information.

**Table 4:** DIF for the 11 YFAS2 diagnostic criteria

Diagnostic criteria	*t* value	*P* value	DIF contrast*
* Gender DIF *			
Substance taken in larger amount and for longer period than intended (#1+#2+#3)	− 2.46	0.01	− 0.70
Persistent desire or repeated unsuccessful attempts to quit (#4+#25+#31+#32)	− 1.60	0.11	− 0.49
Much time/activity to obtain, use, recover (#5+#6+#7)	− 1.36	0.18	− 0.48
Important social, occupational, or recreational activities given up or reduced (#8+#10+#18+#20)	2.43	0.02	0.58
Use continues despite knowledge of adverse consequences (e.g., emotional problems, physical problems) (#22+#23)	− 0.19	0.85	− 0.05
Tolerance (marked increase in amount; marked decrease in effect) (#24+#26)	− 1.06	0.29	− 0.34
Characteristic withdrawal symptoms; substance taken to relieve withdrawal (#11+#12+#13+#14+#15)	− 2.39	0.02	− 0.61
Continued use despite social or interpersonal problems (#9+#21+#35)	3.10	0.00	0.78
Failure to fulfill major role obligation (e.g., work, school, home) (#19+#27)	4.68	0.00	1.12^+^
Use in physically hazardous situations (#28+#33+#34)	− 1.97	0.05	− 0.46
Craving, or a strong desire or urge to use (#29+#30)	− 0.94	0.35	− 0.29
*BMI DIF*			
Substance taken in larger amount and for longer period than intended (#1+#2+#3)	− 0.08	0.94	− 0.02
Persistent desire or repeated unsuccessful attempts to quit (#4+#25+#31+#32)	0.63	0.53	0.21
Much time/activity to obtain, use, recover (#5+#6+#7)	− 0.69	0.49	− 0.25
Important social, occupational, or recreational activities given up or reduced (#8+#10+#18+#20)	− 0.91	0.36	− 0.23
Use continues despite knowledge of adverse consequences (e.g., emotional problems, physical problems) (#22+#23)	1.06	0.29	0.32
Tolerance (marked increase in amount; marked decrease in effect) (#24+#26)	− 0.54	0.59	− 0.18
Characteristic withdrawal symptoms; substance taken to relieve withdrawal (#11+#12+#13+#14+#15)	0.57	0.57	0.15
Continued use despite social or interpersonal problems (#9+#21+#35)	− 1.66	0.1	− 0.45
Failure to fulfill major role obligation (e.g., work, school, home) (#19+#27)	− 0.76	0.45	− 0.19
Use in physically hazardous situations (#28+#33+#34)	1.58	0.12	0.39
Craving, or a strong desire or urge to use (#29+#30)	0.84	0.40	0.27

*DIF* differential item functioning

*Positive values indicate that the probability of the item endorsement is easier for male participants than female participants; easier for participants with BMI < 24 than those with BMI ≥ 24

+DIF contrasts over 1 indicate substantial DIF

### mYFAS 2.0

Rasch analyses were also conducted on the mYFAS 2.0. We first examined the modified 13 FA symptoms. All items demonstrated great goodness-of-fit values to the Rasch model with the proper range of MnSq and Zstd values (Table 5), which met the criteria that the mYFAS2.0 had less than 5% of the misfit items. The most adopted item among the 13 symptoms was item #13 “If I had emotional problems because I hadn’t eaten certain foods, I would eat those foods to feel better,” while the least adopted items were tied between item #19 “My overeating got in the way of me taking care of my family or doing household chores” and item #33 “I was so distracted by eating that I could have been hurt (e.g., when driving a car, crossing the street, operating machinery).” The measure logits ranged from − 0.41 to 0.32. Table 5 offers additional details.

**Table 5:** Rasch analyses of the 13 modified YFAS2 (mYFAS2) symptoms

Item		Measure		SE		Infit				Outfit			
						MnSq		Zstd		MnSq		Zstd	
3.I ate to the point where I felt physically ill		− 0.22		0.04		1.38		6.0		1.87		9.0	
5. I spent a lot of time feeling sluggish or tired from overeating.		− 0.09		0.04		0.98		− 0.2		1.11		1.7	
10. I avoided work, school or social activities because I was afraid I would overeat there.		0.17		0.04		0.84		− 2.8		0.77		− 3.5	
13. If I had emotional problems because I hadn’t eaten certain foods, I would eat those foods to feel better.		− 0.41		0.04		1.38		6.2		1.78		9.0	
16. My eating behavior caused me a lot of distress.		− 0.03		0.04		0.92		− 1.3		0.92		− 1.1	
17. I had significant problems in my life because of food and eating. These may have been problems with my daily routine, work, school, friends, family, or health.		0.01		0.04		0.75		− 4.7		0.76		− 3.8	
19. My overeating got in the way of me taking care of my family or doing household chores.		0.32		0.04		0.78		− 3.9		0.63		− 5.8	
22. I kept eating in the same way even though my eating caused emotional problems.		0.04		0.04		1.02		0.3		1.03		0.4	
24. Eating the same amount of food did not give me as much enjoyment as it used to.		0.06		0.04		0.89		− 2.0		0.80		− 3.0	
29. I had such strong urges to eat certain foods that I couldn’t think of anything else.		0.05		0.04		0.75		− 4.6		0.88		− 1.7	
32. I tried and failed to cut down on or stop eating certain foods.		− 0.33		0.04		1.45		7.2		1.76		9.0	
33. I was so distracted by eating that I could have been hurt (e.g., when driving a car, crossing the street, operating machinery).		0.32		0.04		0.88		− 2.1		0.67		− 5.0	
35. My friends or family were worried about how much I overate.		0.11		0.04		1.24		3.8		1.30		4.0	

*MnSq* mean square; *Zstd* standardized score; *SE* standard error

When considering the goodness-of-fit of the diagnostic criteria from the mYFAS 2.0, all 11 met the Rasch expectation (Table 6). The most adopted criterion was “*Important social, occupational, or recreational activities given up or reduced*,” which was the second most in the YFAS2.0. The least adopted criterion was “*Much time/activity to obtain, use, recover*,” which was the same as the result in the YFAS2.0. The measure logits ranged from − 2.26 to 2.16. Please refer to Table 6 for additional details.

**Table 6:** Rasch analyses of the 11 modified YFAS2 (mYFAS2) diagnostic criteria

Diagnostic criteria	Measure	SE	Infit				Outfit		
			MnSq		Zstd		MnSq	Zstd	
Substance taken in larger amount and for longer period than intended (#3)	0.71	0.14	1.13		1.5		2.29	5.1	
Persistent desire or repeated unsuccessful attempts to quit (#32)	1.32	0.16	1.22		2.1		2.97	5.1	
Much time/activity to obtain, use, recover (#5)	2.16	0.18	1.09		0.8		1.33	0.8	
Important social, occupational, or recreational activities given up or reduced (#10)	− 2.26	0.12	0.96		− 0.7		1.23	1.1	
Use continues despite knowledge of adverse consequences (e.g., emotional problems, physical problems) (#22)	0.53	0.14	0.92		− 0.9		1.20	1.1	
Tolerance (marked increase in amount; marked decrease in effect) (#24)	2.00	0.17	0.96		− 0.3		1.82	0.19	
Characteristic withdrawal symptoms; substance taken to relieve withdrawal (#13)	0.14	0.13	1.11		1.4		1.84	4.6	
Continued use despite social or interpersonal problems (#35)	− 2.11	0.12	0.81		− 3.5		0.98	0.0	
Failure to fulfill major role obligation (e.g., work, school, home) (#19)	− 1.68	0.12	0.74		− 4.9		0.71	− 1.9	
Use in physically hazardous situations (#33)	− 1.72	0.12	0.77		− 4.4		1.22	1.3	
Craving, or a strong desire or urge to use (#29)	0.91	0.15	0.72		− 3.5		0.80	− 0.9	

*MnSq* mean square; *Zstd* standardized score, *SE* standard error

The person separation for the mYFAS 2.0 was 2.17 with an acceptable person reliability of 0.82. These values indicated that the mYFAS 2.0 items could distinguish enrolled participants into 3.23 strata of respondents.

No substantial gender- or BMI-related DIF was detected for the mYFAS 2.0. The contrasts for gender (i.e., male vs. female) ranged from − 0.73 to 0.94. The contrasts for BMI status (i.e., BMI ≥ 24 vs. BMI < 24) ranged from − 0.66 to 0.42. Please refer to Table 7 for further details.

**Table 7:** DIF for the 11 mYFAS2 diagnostic criteria

Diagnostic criteria	*t* value	*P* value	DIF contrast*
*Gender DIF*			
Substance taken in larger amount and for longer period than intended	− 0.37	0.71	− 0.11
Persistent desire or repeated unsuccessful attempts to quit	− 2.18	0.03	− 0.69
Much time/activity to obtain, use, recover	− 0.06	0.95	− 0.02
Important social, occupational, or recreational activities given up or reduced	3.77	< 0.01	0.94
Use continues despite knowledge of adverse consequences (e.g., emotional problems, physical problems)	− 0.89	0.38	− 0.25
Tolerance (marked increase in amount; marked decrease in effect)	0.17	0.87	0.06
Characteristic withdrawal symptoms; substance taken to relieve withdrawal	− 2.69	0.01	− 0.73
Continued use despite social or interpersonal problems	− 1.06	0.29	− 0.26
Failure to fulfill major role obligation (e.g., work, school, home)	3.41	< 0.01	0.83
Use in physically hazardous situations	− 0.26	0.79	− 0.06
Craving, or a strong desire or urge to use	− 0.87	0.39	− 0.26
*BMI DIF*			
Substance taken in larger amount and for longer period than intended	0.40	0.69	0.13
Persistent desire or repeated unsuccessful attempts to quit	0.91	0.36	0.31
Much time/activity to obtain, use, recover	− 1.76	0.07	− 0.66
Important social, occupational, or recreational activities given up or reduced	− 1.41	0.16	− 0.37
Use continues despite knowledge of adverse consequences (e.g., emotional problems, physical problems)	0.62	0.53	0.19
Tolerance (marked increase in amount; marked decrease in effect)	− 1.51	0.13	− 0.55
Characteristic withdrawal symptoms; substance taken to relieve withdrawal	0.15	0.88	0.04
Continued use despite social or interpersonal problems	− 0.38	0.70	− 0.10
Failure to fulfill major role obligation (e.g., work, school, home)	0.08	0.93	0.02
Use in physically hazardous situations	1.61	0.11	0.42
Craving, or a strong desire or urge to use	0.71	0.48	0.23

*DIF* differential item functioning

No substantial DIF was found in mYFAS 2.0

*Positive values indicate that the probability of the item endorsement is easier for male participants than female participants; easier for participants with BMI < 24 than those with BMI ≥ 24

## Discussion

This study was designed for the psychometric assessment of the YFAS 2.0 and mYFAS 2.0 using Rasch analysis in terms of item fit and DIF. The current findings indicated that both scales have an acceptable structural validity without considerable DIF, making them appropriate measurements of FA. Also, we found both the diagnostic criteria and raw symptom counts included in the measures would be useful to indicate FA. Comparison between the two scales in term of item fitness indicated that the mYFAS 2.0 compared to its full version (YAFS 2.0) had fewer misfit items and no significant DIF for gender. Additionally, both scales did not show significant DIF related to BMI groupings. Implications are discussed below.

Several studies have investigated the construct validity of the YFAS measures using CTT methods [13, 14, 16, 17]. Koball et al. [12] assessed the dimensionality of the YFAS 2.0 using convergent and discriminant validity methods in patients seeking bariatric surgery and found that the scale appropriately measured FA as a unique construct. Consistent with our findings, they suggested that diagnostic criteria are appropriate for evaluating information obtained from the scale. In another attempt to validate the original YFAS scale that was developed based on DSM-IV criteria, Manzoni and colleagues reported a single-factor model in Italian university students based on results from confirmatory factor analysis [13]. They also, as we found, suggested some revision in several items due to low factor loading and high unusual correlation between a few items. These findings reveal that although the original YFAS scale developed by Gearhardt and colleagues in 2009 seemed to have acceptable psychometric properties [49], the newer versions (i.e., YFAS 2.0 and mYFAS) apparently have more appropriate validity and reliability, suggesting a formative evolution of the scales over time.

Other validations of the YFAS 2.0, including those conducted in college students in China and among primary-care clinic clients in Malaysia [9, 50], likewise confirmed the unidimensionality of the scale, congruent with our findings. However, our analysis has been done with interval values instead of ordinal ones, providing additional confidence in the results. However, Nantha and colleagues, when assessing the internal consistency of the scale using Kuder-Richardson α, found that the YFAS 2.0 had good reliability for both diagnostic-criteria and symptom-count versions [50]. We believe the seemingly conflicting results with ours may be attributed to different kinds of statistical analysis, recruitment of different target groups or other factors. A single factor solution for the mYFAS 2.0 has also been observed in a large sample of Brazilian people and a sample of college students in China using both exploratory and confirmatory factor analyses [9, 20].

Carr et al. [26] performed a study on a sample of university students in the United States to estimate measurement invariance of the YAFS 2.0 based on gender and race/ethnicity variables and found promising results supporting absence of significant invariance. However, they found that a single diagnostic indicator related to “efforts to cut down on tasty food” varied between gender groups. Similarly, we assessed the invariance using DIF and found the diagnostic criterion of the “failure to fulfill major role obligation” may differentiate responses from females and males. The diagnostic-criteria results in that study were different from our findings. This suggests that some diagnostic criteria assessed by the measure may exhibit different patterns based on gender in different people from different cultures and speaking different languages. Therefore, regarding the sensitivity of the gender variable, it is suggested that further evaluation of the criteria in other settings with different statistical methods be planned to detect any further potential DIF in other criteria or among different target groups.

In developing the modified version of the YAFS 2.0, Schulte and Gearhardt reported that both scales demonstrated relatively equivalent psychometric properties when assessing FA in different populations [19]. However, we found that the mYFAS 2.0 may have fewer misfit items and less DIF compared to the full version as captured by the Rasch analysis. Nevertheless, because the full version of the scale with more items may provide more detailed information on the 11 diagnostic criteria of FA, we recommend using the YFAS 2.0 when wishing to obtain thorough information. In contrast, the mYFAS 2.0 could be used in busy clinical settings. That is, we believe both scales are appropriate instruments, and their use should be based on the desired purposes of reliably gathering more information with greater respondent burden (using the YFAS 2.0) or less information with less respondent burden (using the mYFAS 2.0). Thus, multiple options exist given that both instruments are psychometrically sound.

Although we believe using a modern statistical method (namely Rasch modeling) and computing DIF may distinguish our study from former assessments of the psychometric properties of the scales, there are several study limitations that should be mentioned. First, we conducted the study in university students and faculty members without any diagnosed nutritional or psychological disorders. Because FA has been linked to other disorders, assessing the properties among in other participants, particularly those with eating disorders or addictive behaviors, may generate different findings, and this needs further study. Second, social desirability among educated people participating in the study may have influenced the gathered data (for example, with respect to response or desirability biases). Therefore, replication of the study among other people, including those with lower levels of education, is suggested. Third, we used the Chinese versions of the scales. Given potential cultural influences, further investigation of the scales in other countries and languages is needed to evaluate whether the scales may produce similar findings in other populations. Fourth, we chose an online platform to collect data. Although such platforms may help investigators collect data in a timely fashion, individuals who are not members of social networks or without access to internet and online services may not be represented. Therefore, the findings may not be generalizable to these groups. Using multiple methods, such as face-to face data collection and telephone interviews along with online surveys, may address this limitation. Another limitation may be related to sampling bias when some individuals may systematically have greater chance to be included through online recruitment particularly those individuals who are accessible during sampling. Finally, the security of online data acquisition as was done here using *Google Forms* requires further assessment. Based on the available information, *Google* uses strict security protocols to protect information. Nonetheless, this approach to data collection is a potential concern and limitation.

## Conclusion

In conclusion, our study showed both the YFAS 2.0 and its modified version with single factor structures may be recommended as valid and reliable instruments to collect data on FA. Diagnostic criteria included in these scales are suitable indicators to capture information on various aspects of FA, and these criteria may provide more accurate data than symptom counts. Further investigation of the scales in people with food-related disorders or other psychiatric conditions or from other settings and cultures will contribute to a better understanding of how these measures may function in different settings to collect valid information on FA. Healthcare providers, including nutritionists, may benefit from using these scales to assess responses to interventions to treat addictive-like food consumption and investigate the predictive validity of the measures in clinical practice.

## Data Availability

The datasets generated during and/or analyzed during the current study are available from the corresponding author on reasonable request.
